# Case Report: Coexisting cardiac cavernous hemangioma and sarcoidosis in a single lesion: a first reported case confirmed by surgical resection

**DOI:** 10.3389/fcvm.2025.1707426

**Published:** 2025-10-28

**Authors:** Chuan-Yong Xiao, Wen-Ya Han, Han-Sheng Wang, Xin Qian, Ling-Ling Yuan, Mei-Fang Wang, Yu-Quan Liu

**Affiliations:** ^1^ Department of Pulmonary and Critical Care Medicine, Taihe Hospital of Shiyan, Hubei University of Medicine, Shiyan, Hubei, China; ^2^ Shiyan Key Laboratory of Major Chronic Respiratory Disease, Taihe Hospital, Hubei University of Medicine, Shiyan, Hubei, China; ^3^ Department of Pathology, Taihe Hospital, Hubei University of Medicine, Shiyan, China; ^4^ Department of General Practice, Taihe Hospital, Hubei University of Medicine, Shiyan, Hubei, China

**Keywords:** cardiac hemangioma, cardiac sarcoidosis, VEGF, cardiac surgery, hormone

## Abstract

Cardiac hemangioma (CH) is a rare benign cardiac tumor whose clinical manifestations—including arrhythmias, heart failure, and pericardial effusion—vary by anatomical location. Sarcoidosis is a multisystem disorder of unknown etiology characterized by non-caseating granulomas, commonly involving the lungs, eyes, and skin, with cardiac involvement being relatively uncommon. To date, no cases of concurrent cardiac hemangioma and cardiac sarcoidosis have been reported worldwide. We herein describe a patient with established sarcoidosis who, during follow-up, exhibited progressive enlargement of a cardiac mass. Surgical resection confirmed the co-existence of cardiac hemangioma and cardiac sarcoidosis within the same anatomical region. Based on these findings, we propose a pathophysiological mechanism wherein cardiac sarcoidosis causes microvascular injury, leading to structural alterations that may promote the development of cardiac hemangioma. The following report details the diagnostic and therapeutic course of this patient.

## Introduction

Cardiac tumors demonstrate an incidence of approximately 0.001%–0.3% in autopsy series, with cardiac hemangiomas (CH) accounting for about 2.8% of all resected cardiac tumors. CHs are histologically classified into cavernous, capillary, and arteriovenous malformation subtypes, among which the cavernous type constitutes approximately 58.5% of cases (1, 2) The pathogenesis of CH remains incompletely elucidated, though studies have identified dysregulation of the vascular endothelial growth factor (VEGF) and VEGF receptor (VEGFR) signaling pathway in vascular tumors and malformations (3), suggesting a potential association with vascular endothelial injury. Sarcoidosis is a multisystem disorder characterized by non-caseating granulomas involving the lungs, skin, liver, spleen, and heart. Cardiac involvement is identified in 25% of autopsy-confirmed sarcoidosis cases but is clinically apparent in only 5% of patients (4). Notably, elevated VEGF levels have been reported in both bronchoalveolar lavage fluid and serum of sarcoidosis patients compared with healthy controls (5, 6). The prognosis of cardiac sarcoidosis exhibits significant geographic variation, with studies reporting 5-year survival rates ranging between 60% and 90% among Japanese patients, where cardiac involvement accounts for approximately 85% of sarcoidosis-related mortality. In contrast, cardiac manifestations contribute to 13%−25% of sarcoidosis-related deaths in the United States (7).

We present the first documented case of concurrent cardiac hemangioma and cardiac sarcoidosis, in which VEGF may contribute to disease progression. This observation supports a novel hypothesis that cardiac sarcoidosis induces vascular endothelial injury, leading to pathological angiogenesis and subsequent hemangioma formation. However, the underlying molecular mechanisms remain unclear, and this case may provide insights into sarcoidosis-related vascular injury.

## Case-presentation

**Figure 1:**
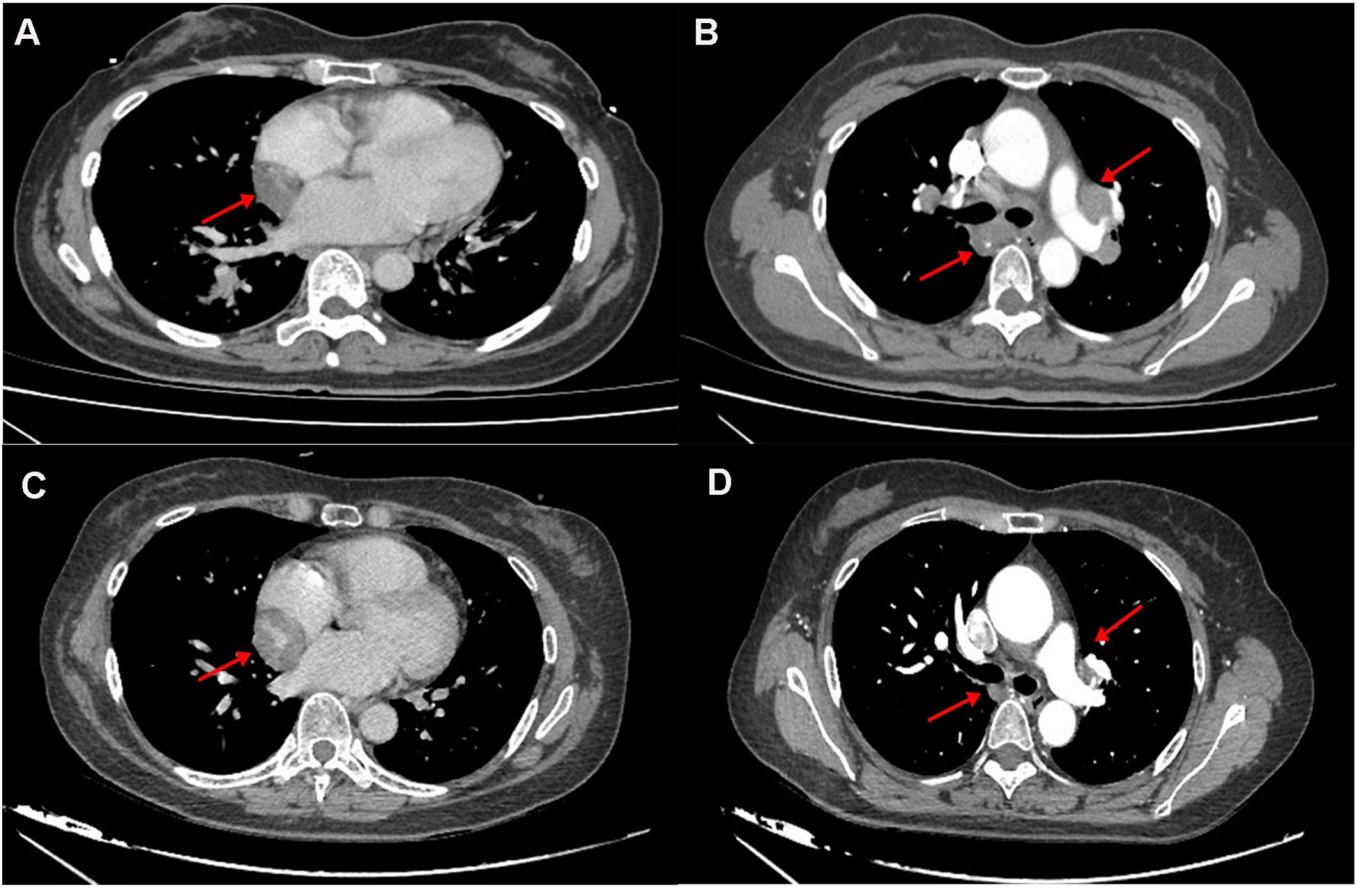
**(A,B)** the 2022 chest CT revealed a hypodense lesion in the interventricular groove and hilar lymphadenopathy. **(C,D)** The 2025 CT revealed progressive enlargement of a mass within the atrioventricular groove (measuring 3.3 cm vs. 4.1 cm in 2022), with concurrent regression of bilateral hilar lymphadenopathy.

A 54-year-old woman presented with incidental findings on a 2022 surveillance chest computed tomography (CT), revealing pulmonary nodules and mediastinal lymphadenopathy alongside a 3.3 × 2.4 cm lesion at the atrioventricular groove (Figures 1A,B). Subsequent integrated positron emission tomography-computed tomography (PET-CT) suggested potential neoplastic pathology. Diagnostic confirmation of pulmonary sarcoidosis was established via endobronchial ultrasound-guided transbronchial needle aspiration (EBUS-TBNA) of mediastinal lymph nodes and percutaneous lung biopsy in 2022. The patient initiated protocol-based oral methylprednisolone therapy with gradual tapering, achieving discontinuation after 18 months. Six months post-discontinuation, surveillance imaging demonstrated recurrent mediastinal lymphadenopathy, prompting reinitiation of methylprednisolone, which induced lymph node regression. Long-term oral methylprednisolone (2 mg daily) was initiated as maintenance therapy. Throughout this period, the atrioventricular groove lesion exhibited no significant interval changes.During an April 2025 follow-up, transthoracic echocardiography detected a solid mass at the right atrial roof demonstrating interval progression compared to prior studies. The patient remained asymptomatic without palpitations, chest tightness, or dyspnea. Medical history was negative for hypertension, coronary artery disease, or tobacco use. At the time of physical examination, the patient was afebrile. His heart rate was 82 beats/min, respiratory rate was 18 breath/min, and blood pressure 108/78 mmHg, SpO_2_ was 98% (room air). Cardiopulmonary examination was unremarkable, with no evidence of jaundice, petechiae, or scleral icterus on integumentary and ocular inspection; no palpable lymphadenopathy was detected in superficial nodal basins, and bilateral eyelids were symmetric without visual disturbances.

**Figure 2:**
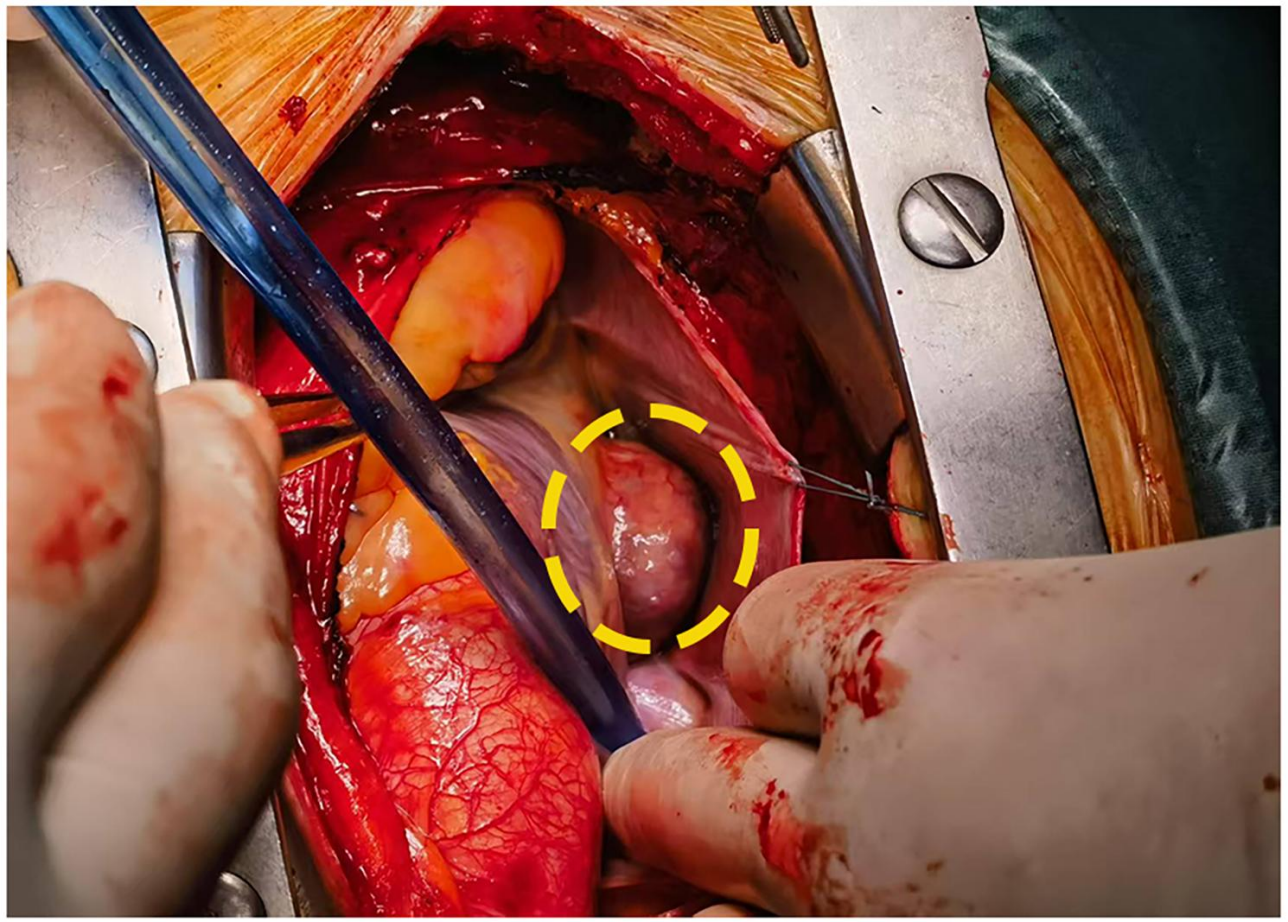
Intraoperative visualization confirmed a tumor in the atrioventricular groove(inside the yellow ellipse).

**Figure 3:**
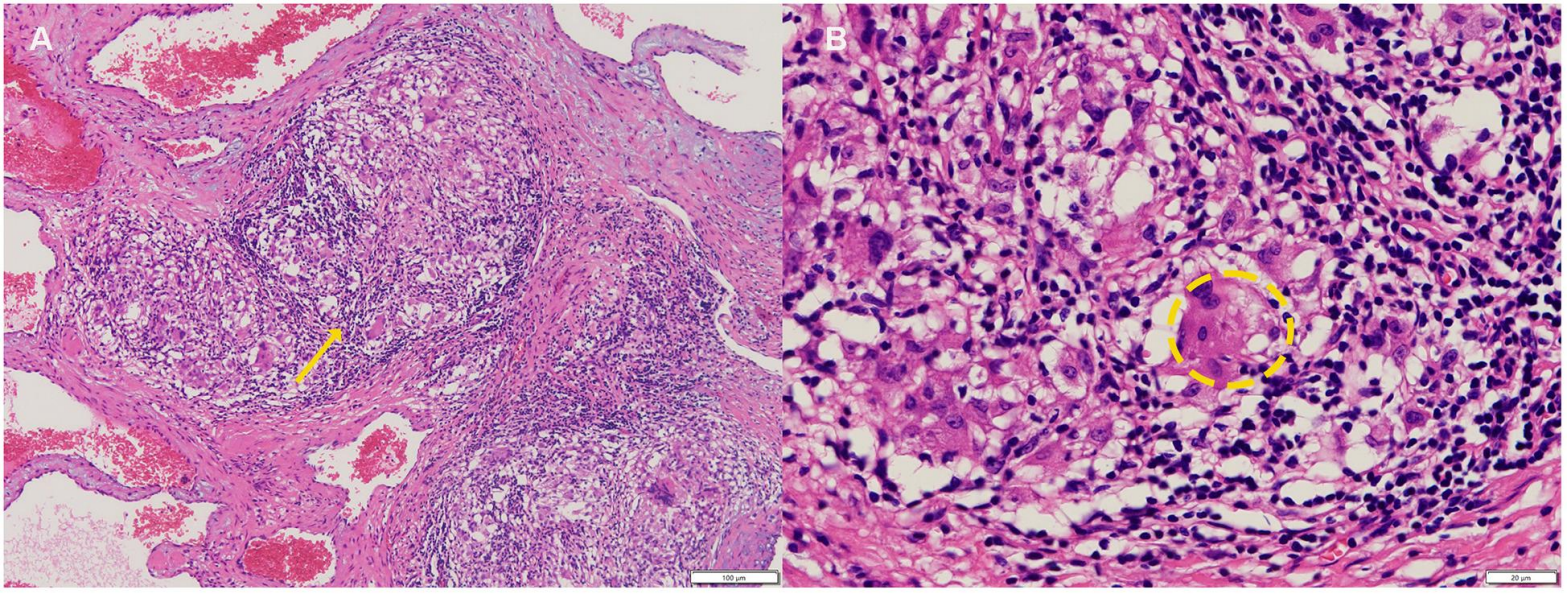
**(A)** Cavernous hemangioma with granulomatous inflammation(yellow arrow). **(B)** Occasionally showing asteroid bodies within multinucleated giant cells (Inside the yellow ellipse,hematoxylin-eosin, 100× original magnification).

Laboratory studies revealed mild elevation of gamma-glutamyl transferase with otherwise unremarkable findings as detailed in Table 1. Contrast-enhanced chest computed tomography (CT) demonstrated a well-demarcated 4.1 × 2.7 cm mass adjacent to the right atrial roof, exhibiting a mean attenuation of 38 Hounsfield units (Figures 1C,D). 24 h ambulatory electrocardiography documented occasional atrial and ventricular premature complexes accompanied by transient horizontal ST-segment depression (0.05 mV in leads II, III, aVF, V5, V6). The right atrial mass revealed refractoriness to glucocorticoid therapy on retrospective chest CT review and exhibited interval progression on recent surveillance imaging, its clinical behavior was incompatible with a singular CS pathology. To achieve definitive histopathological diagnosis and guide subsequent therapeutic intervention, multidisciplinary shared decision-making with the patient and family culminated in proceeding with surgical resection. Preoperative coronary angiography showed no significant stenosis, while transesophageal echocardiography (TEE) revealed a 38 × 29 × 27 mm hypoechoic mass at the right atrial roof with distinct borders, broad-based attachment, regular contours, and absence of internal vascular flow. On May 28, 2025, cardiac tumor resection under general anesthesia was performed. Intraoperative exploration identified an intrapericardial mass (30 × 25 mm) with firm consistency, transversing the interatrial groove and exhibiting firm adhesions to bilateral atrial walls (Figure 2). Postoperative TEE confirmed complete resection without evidence of residual mass. Histopathological analysis of the excised atrial mass identified a cavernous hemangioma with granulomatous inflammation (Figure 3). Special staining analyses demonstrated comprehensively negative results across all performed assays: Periodic acid-Schiff (PAS), acid-fast bacilli (AFB) stain, silver stain, and Gram stain showed no pathological reactivity. Additionally, polymerase chain reaction (PCR) testing for Mycobacterium tuberculosis complex yielded negative results, further excluding mycobacterial involvement.

**Table 1:** Routine laboratory findings.

Laboratory test	Results	Reference range
Leucocyte	9.04 × 10^9 ^/L	3.5–9.5 × 10^9 ^/L
Blood platelet	189 × 10^9 ^/L	125–350 × 10^9 ^/L
Hb	139 g/L	115–150 g/L
Red blood cell	4.53 × 10^12 ^/L	3.8–5.1 × 10^12 ^/L
Prothrombin time	11.90 s	9–13 s
Partial thromboplastin time	29.70 s	25–37 s
Fibrin monomer	3.47 g/L	2–4 g/L
Alkaline phosphatase	69 U/L	50–135 U/L
Aspartate aminotransferase	29.4 U/L	0–35 U/L
Alanine aminotransferase	6.6 U/L	0–40 U/L
Gamma-glutamyl transferase	51.3 U/L	0–45 U/L
Creatinine	53.16 umol/L	55–120 umol/L
Urea	4.57 umol/L	1.7–7.5 umol/L
Total bilirubin	13.11 umol/L	0–26 umol/L
Sodium	142.70 mmol/L	137–147 mmol/L
Potassium	3.94 mmol/L	3.5–5.3 mmol/L
Glycosylated hemoglobin	5.48%	3.6%–6%
ACEI	46.27 U/L	24–139 U/L

In light of the patient's documented history of systemic sarcoidosis, the patient was diagnosed with concomitant cardiac cavernous hemangioma and granulomatous sarcoidosis. On June 1, 2025, a postoperative contrast-enhanced chest computed tomography (CT) scan confirmed complete resection of the lesion with residual mild pulmonary inflammation and bilateral pleural effusion. Subsequent management included broad-spectrum antimicrobial therapy, therapeutic thoracentesis for symptomatic relief, proton-pump inhibition for gastric protection, and multimodal analgesia for pain control. The patient maintained hemodynamic stability throughout the recovery period, achieved all discharge criteria, and was discharged uneventfully on postoperative day ten. Following histopathological review at Peking Union Medical College Hospital (PUH), the diagnosis of concomitant cardiac cavernous hemangioma and granulomatous sarcoidosis was definitively confirmed. The patient initiated intensified immunosuppressive therapy consisting of prednisone 20 mg daily and methotrexate 12.5 mg daily for an initial three-month course. The patient remains under structured longitudinal surveillance to assess therapeutic response and disease trajectory.

## Discussion

CH account for approximately 2.8% of all resected primary cardiac tumors, classified histologically into cavernous (58.5%), capillary, and occlusion subtypes (8). While predominantly diagnosed in children and adolescents. CH may occur across all age groups with a male predilection. Most patients remain asymptomatic, with tumors incidentally detected during imaging surveillance. Clinical manifestations critically depend on lesion location and size (9, 10):

1. Epicardial tumors: May compress coronary arteries (causing myocardial ischemia), atrial/ventricular chambers (right atrial compression impeding systemic venous return, manifesting as peripheral edema)
2. Endocardial involvement: Can precipitate congestive heart failure
3. Septal invasion: Associated with atrioventricular conduction blocks and risk of sudden cardiac death
4. Epicardial localization represents the most common presentation.

In a case series of 200 CH patients, the right atrium (26.2%) constituted the primary site, followed by the left ventricle (23.1%); rare cases originate from cardiac valves. Electrocardiographic findings range from nonspecific ST-T abnormalities and normal tracings to conduction disturbances (11). In this patient, 24 h Holter monitoring showed occasional premature complexes and transient nonspecific ST-T changes, attributable to the interatrial groove location without significant atrial compression.

This patient with a histologically confirmed history of sarcoidosis maintained on long-term methylprednisolone therapy remained asymptomatic throughout surveillance. Serial chest CT scans at 3–6 month intervals demonstrated radiographic stability of cardiac lesions.In June 2022, PET-CT showing no metabolic activity in the cardiac mass, warranting continued observation without intervention. During the current follow-up, echocardiography revealed a suspected atrial myxoma, while concurrent CT confirmed unequivocal lesion progression. Following exclusion of surgical contraindications and informed consent acquisition, the patient underwent cardiac lesion resection. Intraoperative exploration identified an epicardial hemangioma traversing the interatrial groove with firm adhesions to bilateral atrial walls—an anatomic presentation discordant with preoperative imaging localization and representing a rare topographic variant. Histopathological analysis confirmed cardiac cavernous hemangioma with granulomatous inflammation. Rigorous special staining excluded fungal, mycobacterial, and atypical pathogens, while contrast-enhanced CT and Doppler echocardiography ruled out thrombus or vegetations. This assessment established a definitive diagnosis of concomitant cardiac hemangioma and sarcoidosis.

CH exhibit exceptional rarity, with comorbidities predominantly limited to heart failure and arrhythmias; non-cardiac comorbidities remain exceedingly uncommon. Xie et al. documented a singular case of cardiac hemangioma coexisting with rheumatic heart disease, infer that the reason for the formation of cardiac hemangioma is that, after local necrosis of cardiac tissue caused by rheumatism, the blood vessels dilate to form a cavity, and the surrounding blood vessels dilate to form hemangioma (12). Sarcoidosis, akin to rheumatological disorders, demonstrates multisystem organ involvement. However, vascular infiltration is not an established feature of this disease. Published reports document associations with aortitis and coronary artery involvement, the latter manifesting as reduced coronary flow reserve (13, 14). Particularly, Seref et al. demonstrated patients with sarcoidosis had lower coronary flow velocity reserve(CFVR) compared to healthy controls, thus suggesting a dysfunction in the coronary microvasculature. A reduced response to vasodilators suggests possible structural alterations of the myocardial microvasculature (15). Based on the postoperative histopathological findings of cavernous hemangioma with granulomatous inflammation in this case, we postulate that cardiac sarcoidosis may have induced microvascular structural alterations, potentially leading to hemangioma formation through VEGF-mediated pathological angiogenesis, given the established association between sarcoidosis and elevated VEGF expression. As a single-case report lacking immunohistochemical validation, this study cannot establish a definitive causal relationship between cardiac hemangioma and cardiac sarcoidosis, necessitating further verification through longitudinal follow-up and multicenter clinical data.

Regarding the management of cardiac sarcoidosis, the 2024 American Heart Association guidelines recommend that individuals with persistent or recurrent inflammation following corticosteroid therapy should receive second-line agents (including methotrexate, mycophenolate mofetil, azathioprine, or leflunomide) in combination with corticosteroids. Should follow-up FDG-PET demonstrate evidence of ongoing inflammation, tumor necrosis factor-α targeted therapy with infliximab or adalimumab may be considered as third-line treatment (16). In the present case, the patient exhibited favorable response to glucocorticoids with regression of pulmonary and mediastinal involvement, while the nodular focus at the atrioventricular groove remained stable long-term. However, subsequent surveillance during low-dose maintenance therapy revealed interval progression of the interatrial groove lesion. Although surgical resection was performed, second-line immunosuppressive therapy was initiated accordingly. Should follow-up FDG-PET confirm persistent inflammatory activity, escalation to third-line therapy will be considered.

In summary, we present the first documented case of co-located cardiac hemangioma and sarcoidosis, revealing potential sarcoidosis-induced microvascular alterations and discussing the possible involvement of VEGF-mediated pathogenic mechanisms, while acknowledging study limitations and highlighting the need for clinical vigilance regarding the diverse cardiac manifestations of sarcoidosis.

## Data Availability

The original contributions presented in the study are included in the article/Supplementary Material, further inquiries can be directed to the corresponding author.
